# Establishment and Characterization of Testis Organoids with Proliferation and Differentiation of Spermatogonial Stem Cells into Spermatocytes and Spermatids

**DOI:** 10.3390/cells13191642

**Published:** 2024-10-02

**Authors:** Dong Zhang, Wencong Jin, Yinghong Cui, Zuping He

**Affiliations:** 1 Key Laboratory of Model Animals and Stem Cell Biology in Hunan Province, Hunan Normal University School of Medicine, Changsha 410013, China; zd17831117165@163.com (D.Z.); chillcool_king@163.com (W.J.); yinghongcui@hunnu.edu.cn (Y.C.); 2 Research Center of Reproduction and Translational Medicine of Hunan Province, Changsha 410008, China; 3 Manufacture-Based Learning & Research Demonstration Center for Human Reproductive Health New Technology of Hunan Normal University, Changsha 410013, China

**Keywords:** testicular single cells, transplantation, self-assembly, testis organoids, mitosis and meiosis

## Abstract

Organoids play pivotal roles in uncovering the molecular mechanisms underlying organogenesis, intercellular communication, and high-throughput drug screening. Testicular organoids are essential for exploring the genetic and epigenetic regulation of spermatogenesis in vivo and the treatment of male infertility. However, the formation of testicular organoids with full spermatogenesis has not yet been achieved. In this study, neonatal mouse testicular cells were isolated by two-step enzymatic digestion, and they were combined with Matrigel and transplanted subcutaneously into nude mice. Histological examination (H&E) staining and immunohistochemistry revealed that cell grafts assembled to form seminiferous tubules that contained spermatogonial stem cells (SSCs) and Sertoli cells, as illustrated by the co-expression of PLZF (a hallmark for SSCs) and SOX9 (a marker for Sertoli cells) as well as the co-expression of UCHL1 (a hallmark for SSCs) and SOX9, after 8 weeks of transplantation. At 10 weeks of transplantation, SSCs could proliferate and differentiate into spermatocytes as evidenced by the expression of PCNA, Ki67, c-Kit, SYCP3, γ-HA2X, and MLH1. Notably, testicular organoids were seen, and spermatids were observed within the lumen of testicular organoids after 16 weeks of transplantation, as shown by the presence of TNP1 and ACROSIN (hallmarks for spermatids). Collectively, these results implicate that we successfully established testicular organoids with spermatogenesis in vivo. This study thus provides an excellent platform for unveiling the mechanisms underlying mammalian spermatogenesis, and it might offer valuable male gametes for treating male infertility.

## 1. Introduction

Organoids are three-dimensional (3D) structures that resemble organs, which can be generated through the cultivation of single cells derived from a specific tissue. Organoids actually assume the original structures and functionalities of their respective organs [1,2,3]. Notably, organoids have wider applications in uncovering the molecular mechanisms underlying the development of organs under physiological and pathological conditions [4,5,6]. In the 1970s, cells could be cultured to form organoids, which became a significant milestone in the field of regenerative medicine [7]. Currently, much progress has been achieved in human organoids, e.g., the liver, lungs, intestines, and brain. Nevertheless, there is a lack of studies focusing on reproductive organoids [3,8,9,10,11]. In 2023, the global prevalence of infertility was estimated to be around 17.5% by the World Health Organization (WHO) [12]. According to the ‘China infertility status research report’ issued by The National Health and Family Planning Commission of China, there are around 60 million patients with reproductive issues. The reproductive system comprises male and female reproductive organs [13]. The female reproductive system primarily comprises the ovarian, endometrial, fallopian tube, and trophoblast organs [14,15,16,17]. The first functional human ovarian organoids were successfully generated, and they are capable of secreting sex hormones and facilitating the maturation of reproductive cells and follicle formation [18]. Male reproductive system organoids encompass the testicular, epididymal, and prostate organoids. Testicular organoids can offer valuable gametes for azoospermia and make it feasible to unveil the molecular mechanisms underlying spermatogenesis and male infertility [19,20,21,22]. In 2020, fetal gonad cells were utilized to generate testicular organoids that produce functional spermatids in vitro [23]. Ex vivo testicular organoids might offer an approach to preserving male fertility for pre-pubescent patients with cancer. The key feature of organoid generation lies in the communications of germline stem cells and somatic cells, aiming to mimic the functions of vital tissues and organs in vivo [24,25].

Testicular organoids, by definition, refer to organoids that involve the processes of the dissociation and digestion of testicular tissues by enzymes into single cells, which is followed by their self-assembly into 3D organoids that mimic the structures and functions of in vivo testes. This model comprises various kinds of cell types, including spermatogonial stem cells (SSCs), Sertoli cells, peritubular myoid cells, and mesenchymal cells [26]. The techniques utilized for testicular organoid formation primarily consist of in vivo transplantation and in vitro cultivation. Transplantation is accomplished by subcutaneously grafting testicular cell mixtures into immunodeficient mice [27,28,29,30,31]. Pig testicular cells have been xenotransplanted into nude mice under the skin [32]. Techniques for in vitro cultivation are diverse for generating testis organoids [33]. In 2018, a three-layer gradient system (3-LGS) was developed by sandwiching a matrix containing rat primary testicular cells between two layers of a cell-free matrix. This culture system successfully reconstructed testicular cells into rat testicular organoids, which possessed a functional blood–testicular barrier (BTB) and effectively supported the development of germ cells [34]. Testicular organoids exhibit vascular, tubular, and mesenchymal structures, and they can secrete testosterone [35]. Recently, microfluidic chip-based technology for immature mouse testicular cells was used to form testicular organoids capable of differentiating into meiotic and haploid germ cells [36]. However, the mechanisms underlying the formation of testis organoids remain to be explored.

The period from 0 to 6 days after the birth of mice is characterized by the establishment of the initial pool of SSCs and progenitor cells [37], and ID4^+^ SSCs from P0–6 mice can self-renew without undergoing differentiation [38], reflecting that the SSCs of neonatal mice exhibit great plasticity. In this study, we isolated testicular tissue cells from 5–6-day-old neonatal mice to enrich SSCs, and we mixed these single cells with Matrigel and transplanted them into the subcutaneous regions of nude mice for periods of 8, 10, 12, 14, and 16 weeks. Significantly, we observed the formations of testicular organoids containing viable seminiferous tubules capable of inducing the differentiation of SSCs into spermatocytes and spermatids. The growth of the testis organoids at various stages was compared with normal testes using H&E staining, immunohistochemistry, RT-PCR, and Western blots. Together, we successfully generated testicular organoids with full spermatogenesis in vivo. As such, our study lays a solid basis for exploring molecular mechanisms that mediate mammalian spermatogenesis, and it can provide functional spermatids for treating azoospermia.

## 2. Materials and Methods

### 2.1. Testis Tissue Collection from Mice

Testicular cells were derived from testicular tissues of 5- or 6-day-old ICR (Institute of Cancer Research, Changsha, Hunan, China) mice, while the control testicular tissues were obtained from normal 8-, 10-, 12-, 14-, and 16-week-old ICR mice. All testicular tissues underwent the removal of the capsules and connective tissues. Prior to subsequent experimental procedures, the testicular tissues were washed three times with PBS (phosphate-buffer saline, Hyclone, Grand Island, NY, USA) containing 4% antibiotics with penicillin and streptomycin (Gibco, Grand Island, NY, USA). The mice used in this study were approved by the Biomedical Ethical Committee of Hunan Normal University (No. 2021227).

### 2.2. Isolation of Mouse Testicular Cells

The separation of testicular cells from neonatal mouse testicular tissues was performed using a two-step enzymatic digestion method. The testis tissues were digested with 5 mL DMEM (Dulbecco’s Modified Eagle Medium, Gibco, Grand Island, NY, USA) containing 1 mg/mL collagenase IV (Gibco, Grand Island, NY, USA) and 200 μg/mL DNase I (Sigma, Saint Louis, MO, USA). After a 10 min digestion in a water bath at 34 °C on a shaker, the digestion process was terminated by adding 10 mL DMEM containing 10% FBS (fetal bovine serum, Gibco, Grand Island, NY, USA). Subsequently, the supernatant cells (i.e., Leydig cells and interstitial cells) and sediment (i.e., seminiferous tubules) were separated through centrifugation at 800 rpm for 3 min. The supernatant cells were then filtered using a 40 μm filter. A second enzymatic digestion was conducted with seminiferous tubules using DMEM containing 4 mg/mL collagenase IV, 2 mg/mL hyaluronidase (Sigma, Saint Louis, MO, USA), and 2 mg/mL trypsin (Sigma, Saint Louis, MO, USA) for 5 min in a water bath at 34 °C on a shaker and terminated by the addition of 10 mL DMEM containing 10% FBS. After centrifugation at 800 rpm for three minutes, the supernatant cells (i.e., myoid cells) underwent filtration through a 40 μm nylon mesh, and cell pullets (namely, SSCs and Sertoli cells) were collected and suspended in DMEM/F12 (Dulbecco’s Modified Eagle Medium/Nutrient Mixture F-12, Gibco, Grand Island, NY, USA) medium. The resulting supernatant cells from both digestions were combined and subjected to centrifugation at 1000 rpm for 5 min to obtain the cell sediment.

### 2.3. Reconstructing Testicular Organoids via Subcutaneous Cell Transplantation

The single testicular cells obtained through a two-step enzyme digestion was resuspended in DMEM supplemented with 10% FBS. Subsequently, the cell suspension was combined with Matrigel (Incorporated, Corning, NY, USA) at a 1:1 ratio. The resulting mixture was subcutaneously transplanted into the limbs of nude mice, with an injection of 0.5–1.5 × 10^6^ cells per site. The transplanted cell concentrations were set at 2 × 10^7^ cells/mL, 4 × 10^7^ cells/mL, and 8 × 10^7^ cells/mL, while graft durations ranged from 8 weeks to 16 weeks. Upon completion of each respective cycle, the nude mice were euthanized, and their reconstructed testicular organoids were collected.

### 2.4. Hematoxylin and Eosin (H&E) Staining

Sections of testis organoids and organs were dewaxed at 65 °C for 30 min, followed by incubation with xylene I and II (Fuyu Chemical, Tianjin, China) for 10 min each, anhydrous ethanol (Fuyu Chemical, Tianjin, China) twice for 5 min, and dehydration in graded ethanol (90%, 80%, and 70%) (Fuyu Chemical, Tianjin, China). They were washed three times with distilled water, H&E staining was conducted for 15 min, and sealing with neutral gum (Bioseth, Jiangsu, China) was performed after two rounds of xylene dehydration.

### 2.5. Immunohistochemical Staining

Testicular paraffin sections of organoids and organs were 1–2 μm thick. After the testicular sections were dewaxed with turpentine and gradient concentrations of alcohol, heated in boiled sodium citrate solution for antigen repair, and then blocked with 5% BSA (bovine serum albumin, Sigma, Saint Louis, MO, USA). After being incubated with 1:50 diluted primary antibodies for 12 h at 4 °C, the sections were washed with PBS three times. The primary antibodies included anti-PLZF, anti-SOX9, anti-UCHL1, anti-VASA, anti-CYP17A1, anti-ACTIN, anti-HSD3β, anti-SYCP3, anti-MLH1, anti-γ-H2AX, anti-ACROSIN, and anti-TNP1. The sections were incubated with secondary antibodies at a dilution of 1:1000 using Alexa fluor 488 donkey anti-rabbit lgG or Alexa fluor 594 donkey anti-mouse IgG for 1 h at room temperature. After washing with PBS three times, the nuclei of cells were stained with DAPI. Finally, the images were captured with a fluorescence microscope. Detailed information on the primary and secondary antibodies is shown in Tables S1 and S2.

### 2.6. Extraction of RNA and Reverse Transcription and Polymerase Chain Reaction (RT-PCR)

After homogenizing testicular organoids or testicular organs in Trizol (Vazyme, Jiangsu, China), they were subjected to chloroform (Sevenbio, Beijing, China) extraction, followed by isopropanol and anhydrous ethanol (Fuyu Chemical, Tianjin, China) precipitation. Subsequently, RNA was isolated and dissolved in DEPC-treated water (Beyotime Biochemistry, Changsha, Hunan, China). The concentrations and quality of total RNA were determined using Nanodrop 2000 (Thermo Fisher Scientific, Waltham, MA, USA), and the ratio of A_260_/A_280_ was 1.8–2.0. The One-Step cDNA Synthesis Super Mix (TransGen Biotech, Beijing, China) and the MiniAmp Therma Cycler (Thermo Fisher Scientific, Waltham, MA, USA) were utilized for reverse transcription (RT) to convert mRNA into cDNA. A PCR reaction for 35 cycles was carried out with initial denaturation at 95 °C for 5 min, followed by denaturation at 95 °C for 30 s, annealing at a temperature range of 52–60 °C for 30 s, and elongation at 72 °C for a duration of 45 s. Subsequently, the PCR products were incubated at 72 °C for a period of 7 min. Finally, we separated the PCR products by conducting electrophoresis on a 2% agarose gel at 100 V for 40 min. The primer sequences of genes are listed in Table S3.

### 2.7. Western Blots

The testicular organoids and organs were homogenized in RIPA buffer, followed by ice-cold cleavage and treatment with 10 μL/mL PMSF and 10 μL/mL protease inhibitor. The protein was obtained by centrifugation at 12,000 rpm at 4 °C. Protein concentrations were measured using the bicinchoninic acid (BCA) kit (Dingguo, Beijing, China). The electrophoresis of the proteins was performed on a 10% SDS-PAGE gel at a constant voltage of 80 v for 150 min. Subsequently, proteins were transferred onto PVDF membranes under crossflow conditions at a constant current of 300 mA for 120 min. After being blocked for 30 min, the PVDF membrane was then incubated with primary antibodies overnight at 4 °C. Following three washes with TBST (tris-buffered saline containing 0.1% Tween-20, Biosharp, Hefei, Anhui, China) with each washing lasting ten minutes, the membranes were treated with secondary antibodies at room temperature for 60 min and washed again three times with TBST. Subsequently, the Mini Chemiluminescent Imaging and Analysis System (manufactured by Sagecreation, Beijing, China) was utilized in conjunction with the enhanced chemiluminescence kit (provided by Beyotime Biochemistry, Changsha, Hunan, China) to analyze protein levels. The primary antibodies used in this study were anti-PLZF (R&D Systems, Minneapolis, MN, USA), anti-GFRA1 (R&D Systems), anti-THY1 (Abcam, Cambridge, MA, USA), anti-SYCP3 (Abcam), anti-MLH1 (Abcam), and anti-ACTIN (Santa Cruz Biotechnology, Santa Cruz, CA, USA). The secondary antibodies employed were sheep anti-rabbit, sheep anti-mouse, and donkey anti-sheep (Thermo Fisher Scientific, Waltham, MA, USA). Detailed information on primary and secondary antibodies is shown in Tables S1 and S2.

### 2.8. Spermatocyte Spreading of Testicular Organoids

Mouse testicular organoids were incubated with hypotonic extraction buffer (30 mM tris (pH 8.2), 50 mM sucrose, 17 mM citric acid, 5 mM EDTA, 2.5 mM dithiothreitol, and 1 mM phenylmethylsulfonyl fluoride) for 30 min at room temperature. Spermatocytes were released by squeezing with toothless tweezers in 100 mM sucrose, and they were spread on slides with 1% PFA (pH 9.2) (Fuyu Chemical) containing 0.15% Triton X-100 (Beyotime Biochemistry, Changsha, Hunan, China). The cell slides were washed with 0.04% Photo-Flo (KODAK, Long Island City, NY, USA) for 4 min, and they were incubated with primary antibodies SYCP3 (1:100, Abcam, Cambridge, MA, USA) and γ-H2AX (1:80, Millipore, New Bedford, MA, USA) as well as MLH1 (1:50, Abcam) and SYCP3 (1:100, Abcam) overnight at 37 °C. The cell slides were washed three times with ADB (1% normal donkey serum, 2.5% BSA, and 0.005% triton-X-100), and they were incubated with secondary antibodies donkey anti-mouse 488 and donkey anti-rabbit 555 for 90 min at 37 °C. Finally, images of cells were captured under a fluorescent microscope (Leica, Wetzlar, Germany, DM3000).

### 2.9. Statistical Analysis

All data were acquired from a minimum of three experiments and presented as means ± SEM. Statistical analysis was conducted using GraphPad Prism 5.0, with credibility assessed via the unpaired *t*-test, and a *p*-value < 0.05 denoted statistical significance.

## 3. Results

### 3.1. Formation of Seminiferous Tubules in Testicular Organoids Derived from Testicular Cells

Cell concentrations for transplantation were classified into three groups, namely, 2 × 10^7^ cells/mL, 4 × 10^7^ cells/mL, and 8 × 10^7^ cells/mL. We found that testis organoids derived from the transplanted cell concentration of 2 × 10^7^ cells/mL exhibited a reduced population of SSCs within the seminiferous tubules (Figure S1A, left panel), while testicular organoids generated from the transplanted cell concentration of 8 × 10^7^ cells/mL had a decrease in the efficiency of seminiferous tubule formation (Figure S1A, right panel). The concentration of transplanted testis cells at 4 × 10^7^ cells/mL was shown to form the best testis organoids with the proper morphology of male germ cells and Sertoli cells (Figure S1A, middle panel). We also compared the graft growth in recipient nude mice under castration and uncastrated conditions. An enhancement in differentiation of SSCs was observed in the graft when maintained under uncastrated conditions (Figure S1B, left panel) compared to castration (Figure S1B, right panel).

**Figure 1:**
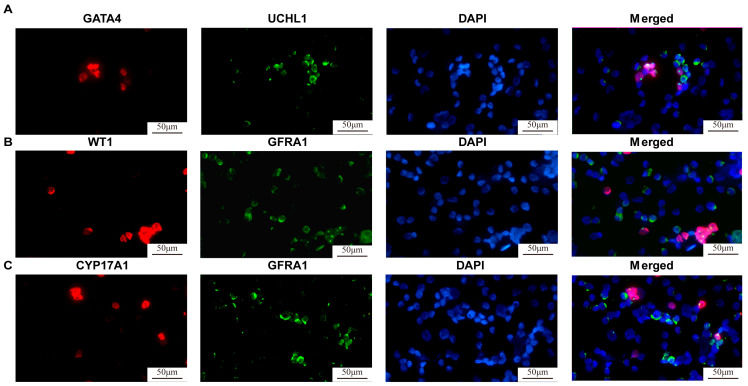
Identification of the isolated testicular single cells. Immunocytochemical staining showed the expression of UCHL1 (**A**), GATA4 (**A**), GFRA1 (**B**,**C**), WT1 (**B**), and CYP17A1 (**C**) in the isolated testicular single cells. DAPI was employed to stain cell nuclei. Scale bars = 50 μm.

**Figure 2:**
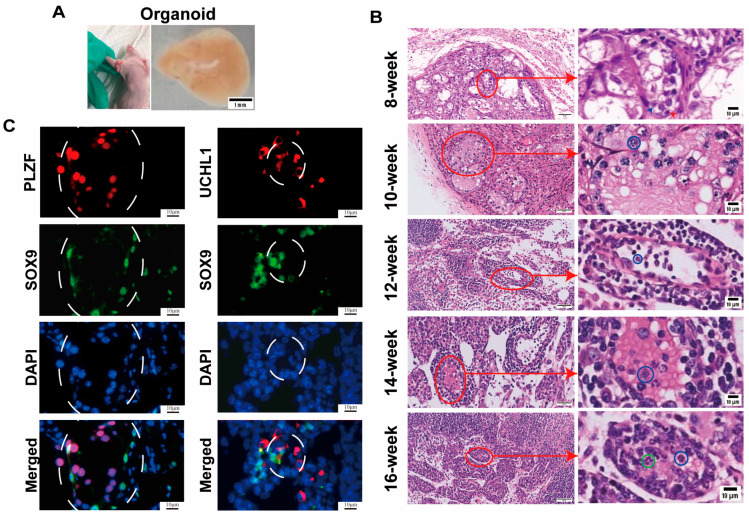
Morphological features of mouse testicular organoids. (**A**) The precise localization of transplantation (left panel) and a representative testicular organoid (right panel) at 8 weeks post-transplantation. Scale bars = 1 mm. (**B**) H&E staining of testicular organoids at 8, 10, 12, 14, and 16 weeks of cell transplantation. The red arrowhead indicates SSCs, while the blue arrowhead denotes Sertoli cells; blue circles indicate spermatocytes, and green circles denote spermatids. Scale bars = 10 μm. (**C**) Double immunostaining of SOX9^+^ Sertoli cells and PLZF^+^ SSCs as well as SOX9^+^ Sertoli cells and UCHL1^+^ SSCs in the 8th testis organoids. White circles denote seminiferous tubules. The red arrows referred to a local zoomed-in image. Scale bars = 10 μm.

Testicular organogenesis occurs during the embryonic/fetal stage through the formation, migration, and assembly of male germ cells and somatic cells [39]. Testicular tissues from neonatal mice at 5–6 days old were isolated as individual cells using a two-step enzyme digestion method, which resulted in a cell mixture containing SSCs and testicular somatic cells. Immunocytochemistry revealed that the isolated testicular single cells were positive for GFRA1 and UCHL1 (Figure 1), hallmarks for SSCs, GATA4 and WT1 (Figure 1), markers for Sertoli cells, and CYP17A1 (Figure 1), a marker for Leydig cells. These data implicate that the isolated testicular single cells comprise SSCs, Sertoli cells, and Leydig cells. These testicular single cells were mixed with a diluted matrix glue solution (matrix glue/culture medium: 1:1), and they were subsequently transplanted into the subcutaneous limbs of nude mice. After 8 weeks of transplantation, the grafts were extracted, and well-defined capsules surrounded the organoid surface (Figure 2A), which was evidence of angiogenesis. Histological examination (H&E) showed that seminiferous tubules underwent development in testicular organs by the 8^th^ week of graft (the red arrowhead indicates SSCs, and the blue arrowhead denotes Sertoli cells) (Figure 2B, first panel). Furthermore, an increased number of pachytene spermatocytes were seen in testis organoids as marked by blue circles at the 10th week (Figure 2B, second panel), and round spermatids were observed as indicated by green circles by the 16th week (Figure 2B, fifth panel). The morphology of seminiferous tubules from normal mouse testis organs at day 6 and 8-16 weeks is shown as reference controls (Figure S2). The cells in the grafts at the 8th week were identified through immunohistochemical staining, showing that PLZF^+^ SSCs (Figure 2C, left panel) and UCHL1^+^ SSCs (Figure 2C, right panel) adhered to the basement membrane and formed tubular structures alongside SOX9^+^ Sertoli cells, which reflects that the formation of seminiferous tubes was established.

**Figure 3:**
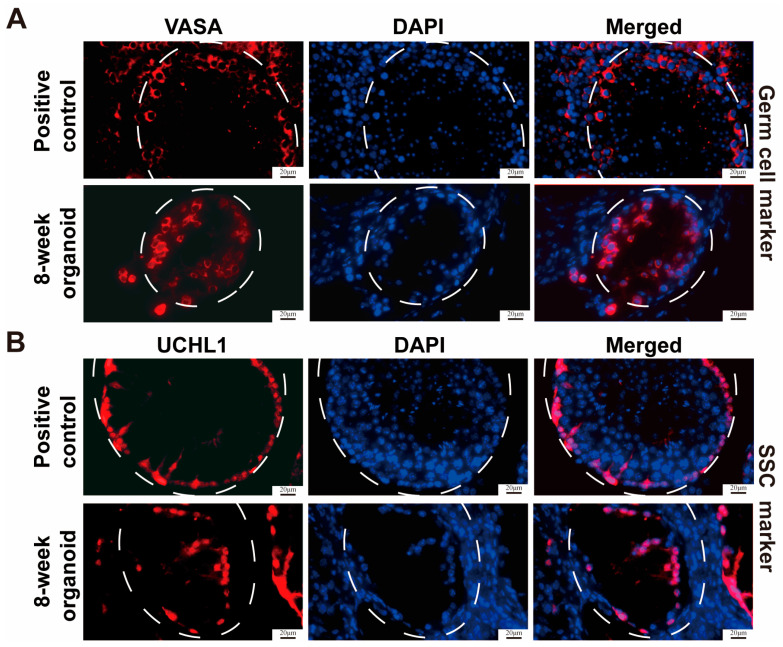
Phenotypic characteristics of germ cells and SSCs in mouse testicular organoids at 8 week of testicular cell transplantation. (**A**,**B**) Immunohistochemical staining revealed the localization of VASA^+^ (**A**) and UCHL1^+^ (**B**) cells within testicular organoids at 8 weeks post-transplantation and control normal testis tissues. White circles indicate seminiferous tubules. Scale bars = 20 μm.

**Figure 4:**
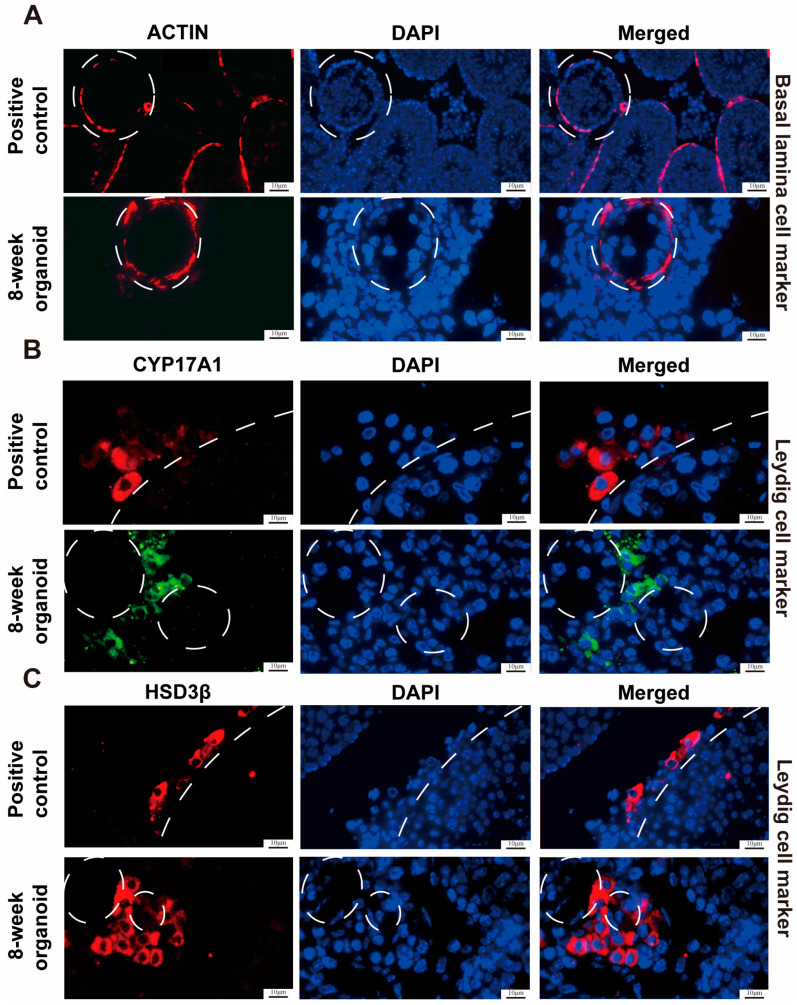
Phenotypic features of somatic cells in mouse testicular organoids at 8 weeks of testicular cell transplantation. (**A**–**C**) Immunohistochemical staining revealed the precise localization of ACTIN-labeled basal lamina cells (**A**), CYP17A1-labeled mesenchymal cells (**B**), and HSD3β-labeled Leydig cells (**C**) in testicular organoids at 8 weeks post-transplantation (lower panels) and normal mouse testis tissues (upper panels). White circles indicate seminiferous tubules. Scale bars = 10 μm.

Immunostaining was performed on the testicular organoids and normal mouse testis organs as positive controls at the 8th week, while IgG replacing the primary antibodies served as a negative control (Figure S3). We found that both testicular organoids and normal mouse testis organs exhibited positive staining for VASA (Figure 3A), a hallmark for germ cells, and UCHL1 (Figure 3B). We also identified the localization of somatic cells in the testicular organoids. The organoid tubules were surrounded by ACTIN^+^ basal lamina cells (Figure 4A), while HSD3B^+^ (Figure 4B) and CYP17A1^+^ Leydig cells (Figure 4C) were distributed among the seminiferous tubules. Based upon the results mentioned above, we have successfully demonstrated the feasibility of reconstructing testicular organoids with SSCs and somatic cells from neonatal mouse testicular cells through subcutaneous transplantation.

### 3.2. SSCs within the Reconstituted Testicular Organoids Possess the Capacity of Proliferation and Differentiation

**Figure 5:**
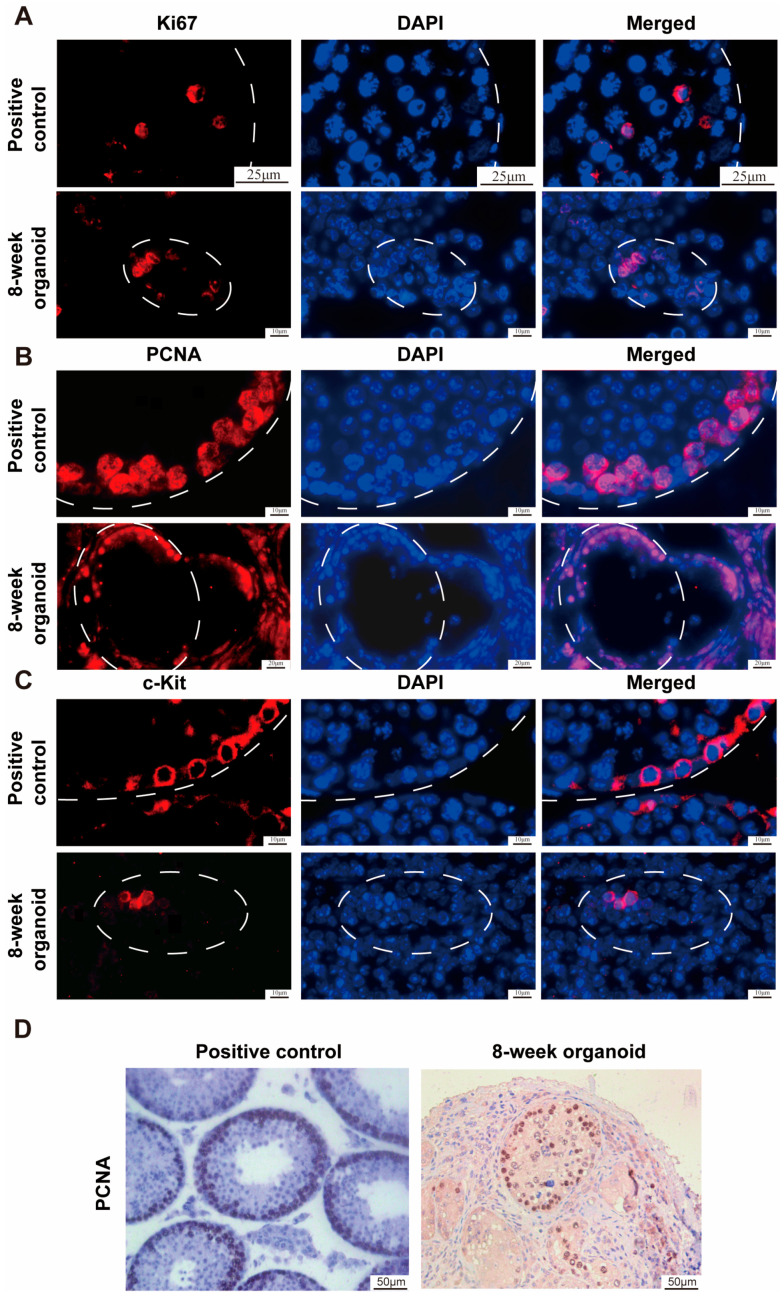
Identification of the proliferation and differentiation capacity of SSCs in the seminiferous tubules of testicular organoids. (**A**–**D**) Immunostaining for Ki67 (**A**), PCNA (**B**,**D**), and c-Kit (**C**) proteins was conducted in 8-week testicular organoids (lower panels) and in the testes of 8-week-old normal mice (upper panels). White circles indicate the seminiferous tubules. Scale bars are shown in the figures.

To investigate the proliferative and differentiation potentials of SSCs within testicular organoids, we conducted immunohistochemical staining on these organoids. SSCs that are positive for PCNA and Ki67 exhibit a capacity for proliferation [40]. We found that Ki67 (Figure 5A, lower panel) and PCNA (Figure 5A, lower panel, and Figure 5D, right panel) were expressed in the SSCs of testicular organoids, and the expression patterns were similar to those in the normal mouse testicular tissues (Figure 5A,B, upper panels, as positive controls). C-Kit is a marker for differentiating spermatogonia, specifically, type A_1_–A_4_ spermatogonia [41]. Our immunostaining revealed that the c-Kit protein was detected in the testicular organoids (Figure 5C, lower panel), which indicates the differentiation of SSCs into type A_1_-A_4_ spermatogonia within the 8th week testicular organoids. As such, our testicular organoids have the capacity to facilitate the proliferation and initial differentiation of SSCs within the seminiferous tubules.

### 3.3. Production of Spermatocytes in Testicular Organoids

**Figure 6:**
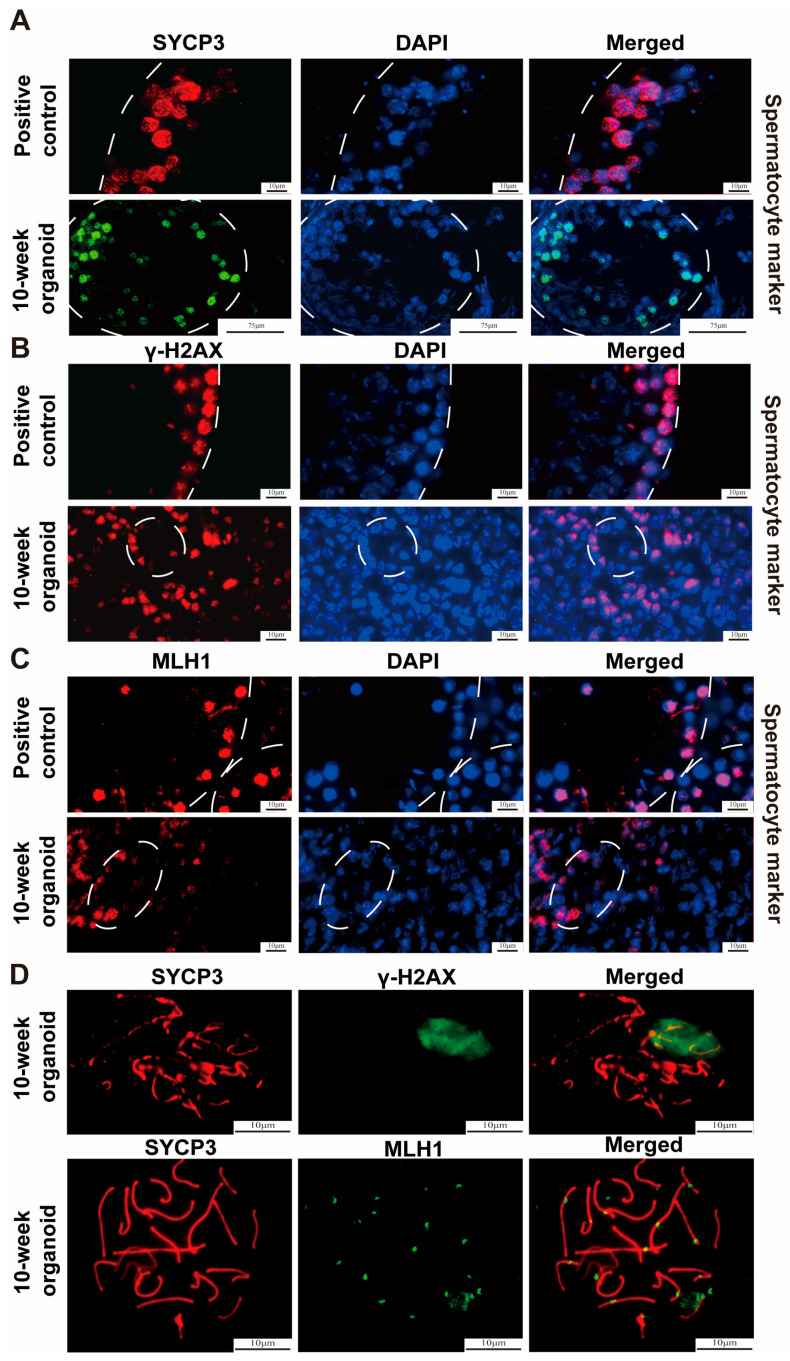
Identification of spermatocytes in the testicular organoids at 10 weeks of testicular cell transplantation. (**A**–**C**) Immunostaining displayed the expression of SYCP3 (**A**), γ-H2AX (**B**), and MLH1 (**C**) proteins in 10-week testicular organoids (lower panels) and in the testes of 10-week-old normal mice (upper panels). White circles denoted seminiferous tubules. (**D**) Spermatocyte spreading and immunostaining displayed the co-expression of SYCP3 and γ-H2AX as well as MLH1 and SYCP3 in 10-week testicular organoids. Scale bars are indicated in the figures.

We found that our testicular organoids at the 8th week of transplantation facilitated the differentiation of SSCs into c-Kit positive type A_1_-A_4_ spermatogonia. Notably, an increased presence of spermatocytes was observed in our testicular organoids at the 10th week of transplantation. H&E staining revealed that spermatocytes exhibited a spherical morphology, with distinct visibility of the chromosomes. SYCP3 protein is a crucial component of the synaptonemal complex, and it plays a vital role in the meiotic process of spermatocytes [42]. MLH1 is an important component of DNA mismatch repair machinery, and it is involved in ensuring accurate meiotic crossovers during the process of meiosis [43]. The phosphorylation of nucleosome variant histone H2AX leads to the formation of gamma-H2AX (γ-H2AX) during the leptotene stage [44]. Significantly, our immunohistochemical staining of SYCP3 (Figure 6A, lower panel), γ-H2AX (Figure 6B, lower panel), and MLH1 (Figure 6C, lower panel) demonstrated the production of leptotene and pachytene spermatocytes within the seminiferous tubules of testicular organoids. Spermatocyte spreading analysis illustrated that SYCP3 and γ-H2AX as well as MLH1 and SYCP3 were expressed in cells of 10-week testicular organoids (Figure 6D). Considered together, these data implicate that SSCs can differentiate into spermatocytes within the seminiferous tubules of the testicular organoids at the 10^th^ week of transplantation.

### 3.4. Generation of Spermatids in Testicular Organoids

**Figure 7:**
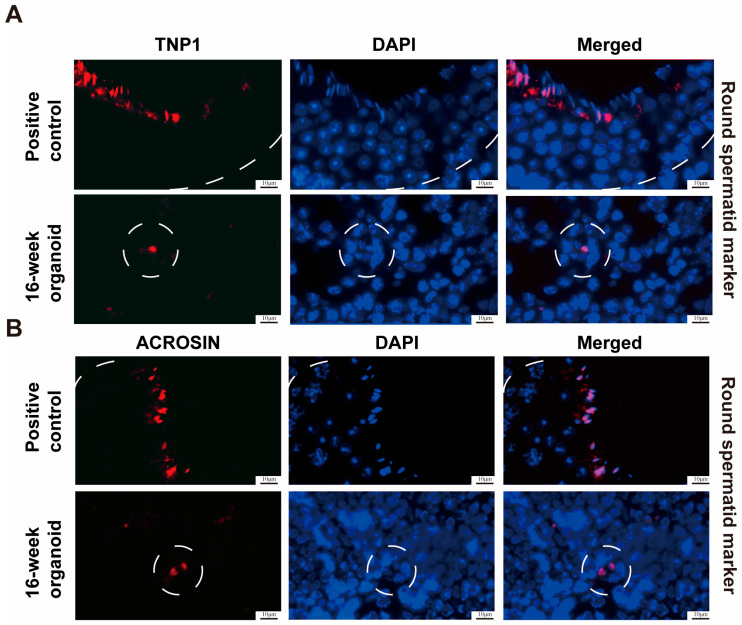
Identification of spermatids in the testicular organoids at 16 weeks of testicular cell transplantation. (**A**,**B**) Immunofluorescence staining illustrated the presence of TNP1 (**A**) and ACROSIN (**B**) in 16-week testicular organoids as well as in the testes of 16-week-old normal mice. White circles denote seminiferous tubules. Scale bars = 10 μm.

To further examine whether the testicular organoids could sustain the continuous differentiation of SSCs into spermatids, we extended the duration of transplantation. A substantial population of spermatocytes was observed at the 10th week of transplantation, while spermatids were detected within the lumen of seminiferous tubules at both the 12th week and 14th week of transplantation (Figure 2B, third and fourth panels). At the 16th week of transplantation, we observed round spermatids exhibiting reduced cellular volumes, spherical nuclei with intense staining, and diminished cytoplasmic content, as indicated by the H&E staining (Figure 2B, fifth panel). Immunostaining illustrated the presence of TNP1 (Figure 7A, lower panel) and ACROSIN (Figure 7B, lower panel) proteins in the seminiferous tubules of the testicular organoids. Together, these results demonstrate that testicular organoids possess a capability for the differentiation of SSCs into round spermatids.

### 3.5. Expression of Testicular Cell Markers in Testicular Organoids

**Figure 8:**
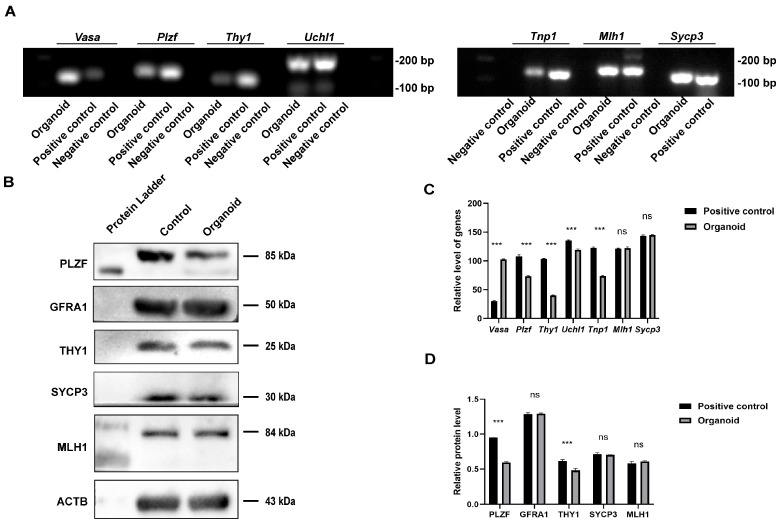
Identification of testicular cell marker genes and proteins in the testicular organoids. (**A**,**C**) RT-PCR revealed the expression of *Vasa*, *Plzf*, *Thy1*, *Uchl1*, *Tnp1*, *Mlh1*, and *Sycp3* genes in the testicular tissue of 16-week-old testicular organoids and normal testis organs of 16-week-old mice. (**B**,**D**) Western Blot showed the expression of PLZF, GFRA1, THY1, SYCP3, and MLH1 proteins in 16-week-old testicular organoids and 16-week-old mouse normal testicular organs. In (**C**) and (**D**), *** denotes a *p*-value < 0.05.

We also assessed the mRNA and protein expression in the testicular organoids at 16 weeks of transplantation using RT-PCR and Western blots. The RT-PCR analysis revealed the presence of germ cells and SSCs, including *Vasa*, *Plzf*, *Thy1*, and *Uchl1*, spermatocyte markers, including *Mlh1* and *Sycp3*, and spermatid hallmarks, e.g., *Tnp1*, in the testicular organoids (Figure 8A,C). Western blotting showed the expression of PLZF, GFRA1, THY1, SYCP3, and MLH1 proteins in these organoids (Figure 8B,D). Statistically, there was no difference in certain gene or protein expression between 16-week testicular organoids and 16-week normal mouse testicular organs (Figure 8A–D).

## 4. Discussion

Generating testicular organoids primarily involves two approaches, namely, the transplantation and in vitro culture of testis cells, with an aim to induce the differentiation of SSCs into spermatids [26]. Subsequent findings reveal the inherent capacity of testicular cells for self-assembly, which enables single testicular cells to form functional testicular tissues [27]. However, the control of transplanted cell concentrations is relatively imprecise, and the recipient nude mice are predominantly castrated [30]. In 2021, porcine testicular organoids have been generated with different concentrations of testis cells [35]. In this study, we investigated three concentrations of transplanted testis cells into the subcutaneous tissues of nude mice. The lower cell concentration at 2 × 10^7^ cells/mL resulted in the formation of loose seminiferous tubules with fewer intercellular connections and a reduced number of SSCs. Meanwhile, the higher cell concentration at 8 × 10^7^ cells/mL led to densely packed cells with limited nutritional supply within the graft and a decreased efficiency in forming seminiferous tubules. Notably, the seminiferous tubules of testis organoids formed by cell transplantation with intermediate concentrations at 4 × 10^7^ cells/mL exhibited enhanced efficiency. The self-renewal and differentiation capacities of SSCs in the testis organoids were influenced by the hormone condition of the recipient nude mice. We observed that the growth of testicular organoids and the differentiation of SSCs were more favorable in recipient nude mice without castration. In contrast, in the castrated mice, there was an increased presence of Sertoli cells within the testicular organoids and a decreased number of SSCs, which resulted in the reduced differentiation of SSCs. In comparison to previous transplantation experiments relying solely on single data verification, this study employed multiple assays, including histological analysis, RT-PCR, immunostaining, spermatocyte spreading, and Western blots, to demonstrate the formation of testicular organoids, as shown the expression of numerous hallmarks for male germ cells, SSCs, spermatocytes, spermatids, and somatic cells including Sertoli cells and Leydig cells. Our findings conclusively imply that testis organoids exhibit seminiferous tubes phenotypically and that SSCs within testicular organoids are able to differentiate into spermatocytes and spermatids.

The construction of testicular organoids in vitro can be achieved through various methods, including both 2D and 3D culture techniques [45]. There are several 3D culture techniques, e.g., the three-layer gradient system, testis-derived scaffolds, and 3D bioprinting [46,47,48]. In comparison to the in vitro culture method, the transplantation approach exhibits enhanced efficiency in organoid construction, facilitates easier operation, and better replicates the in vivo testicular microenvironment or niche. Nevertheless, organoid formation by the transplantation method relies on recipient animals. Consequently, testicular organoids are initially generated through transplantation techniques, followed by comprehensive bioinformatics analysis of RNA-sequencing data to elucidate the pivotal factors involved in testicular organoid development. RNA-sequencing of testicular organoids by KEGG analysis reveals that our testicular organoids possess active pathways involved in intercellular information exchange and intercellular structure formation, thereby highlighting the self-assembly capability of neonatal mouse testicular cells.

## 5. Conclusions

In summary, we have developed a testis organoid formation system capable of supporting the self-renewal and differentiation of SSCs into spermatocytes and spermatids through the subcutaneous transplantation of neonatal mouse testicular cells. This study can offer an excellent platform to unveil molecular mechanisms underlying mammalian spermatogenesis in vivo, and, significantly, it might provide important male gametes for treating azoospermia patients.

## Data Availability

The data are available upon request from the corresponding author.

## Supplementary Materials

The following supporting information can be downloaded at: https://www.mdpi.com/article/10.3390/cells13191642/s1, Table S1: The Detailed Information on Primary Antibodies Used for Immunocytochemistry and Western Blots; Table S2: Secondary Antibodies Utilized in This Study; Table S3: The Sequences of Gene Primers for RT-PCR; Figure S1: H&E staining of testicular organoids at 8 weeks after transplantation under different conditions; Figure S2: The morphology of normal mouse testis organs; Figure S3: The expression of IgG in the mouse testicular tissues.
