# Supplementary material for: Establishment and Characterization of Testis Organoids with Proliferation and Differentiation of Spermatogonial Stem Cells into Spermatocytes and Spermatids
Source: Cells. 2024 Oct 2;13(19):1642. doi: 10.3390/cells13191642 (PMC11476282; doi:10.3390/cells13191642)
Supplement: Supplementary file 1 [file cells-13-01642-s001.zip › cells-3167931-supplementary.pdf]

Establishment and characterization of testis organoids with proliferation and differentiation of spermatogonial stem cells into spermatocytes and spermatids

Dong Zhang, Wencong Jin, Yinghong Cui, Zuping He#

Supplementary Materials

Table S1. The Detailed Information on Primary Antibodies Used for Immunocytochemistry and Western Blots

Antibodies	Vendors	Sources	Working Dilutions
SOX9	Abcam	Mouse	ICC: 1:200
UCHL1	Cell signaling technology	Rabbit	ICC: 1:200
VASA	Abcam	Mouse	ICC: 1:200’
CYP17A1	Santa Cruz Biotechnology	Rabbit	ICC: 1:200
ACTIN	Santa Cruz Biotechnology	Mouse	ICC: 1:200
PLZF	Proteintech	Rabbit	ICC: 1:200/WB 1:1000

HSD3β	Santa Cruz Biotechnology	Rabbit	ICC: 1:200
SYCP3	Abcam	Rabbit	ICC: 1:200/WB 1:1000
MLH1	Abcam	Rabbit	ICC: 1:200/WB 1:1000
γ-H2AX	Millipore	Mouse	ICC: 1:200
ACROSIN	Santa Cruz Biotechnology	Mouse	ICC: 1:200
TNP1	Santa Cruz Biotechnology	Goat	ICC: 1:200
Ki67	Abcam	Rabbit	ICC: 1:200
PCNA	Cell signaling technology	Rabbit	ICC: 1:200
c-Kit	Bio-Techne	Goat	ICC: 1:200
THY1	Abcam	Rabbit	WB 1:1000
GFRA1	Abcam	Goat	WB 1:1000
ACTB	Cell signaling technology	Goat	WB 1:1000

Table S2. Secondary Antibodies Utilized in This Study

Antibodies	Vendors	Catalog Numbers	Working Dilutions
Goat Anti-Mouse HRP	Beyotime Biotechnology	A0126	WB: 1:4000
Goat Anti-Rabbit HRP	Beyotime Biotechnology	A0208	WB: 1:4000
Donkey Anti-Goat HRP	Beyotime Biotechnology	A0181	WB: 1:4000
Anti-Goat Alexa Fluor 488	Thermo Scientific	A11055	ICC: 1:1000
Anti-Goat Alexa Fluor 555	Thermo Scientific	A11058	ICC: 1:1000
Anti-Mouse Alexa Fluor 488	Thermo Scientific	A21202	ICC: 1:1000
Anti-Mouse Alexa Fluor 555	Thermo Scientific	A31570	ICC: 1:1000
Anti-Rabbit Alexa Fluor 488	Thermo Scientific	A21206	ICC: 1:1000
Anti-Rabbit Alexa Fluor 555	Thermo Scientific	A31570	ICC: 1:1000

Table S3. The Sequences of Gene Primers for RT-PCR

Gene Names	Primer Sequences (5' - 3')	Product Sizes (bp)
Vasa	f**orward**: TATGATGCGGGATGGAATAACTG<br>**reverse**: ACAGCTCTTACACAAGTCCCAA	144
Plzf	**forward**：CTGCGGAAAACGGTTCCTG<br>**reverse:** GTGCCAGTATGGGTCTGTCT	150
Thy1	**forward**：TGCTCTCAGTCTTGCAGGTG<br>**reverse:** TGGATGGAGTTATCCTTGGTGTT	121
Uchl1	**forward**：GATGCTGAACAAAGTGTTGGC<br>**reverse:** GGAGTTTCCGATGGTCTGCTT	235
Tnp1	**forward**：ACCAGCCGCAAGCTAAAGAC<br>**reverse**：TTTCCTACTTTTCAGGACGCTC	120
Mlh1	**forward**：GTTTTACTCCATTCGGAAGCAGT<br>**reverse:** TGTGAGCGGAAGGCTTTATAGAT	135
Sycp3	**forward**：AGCCAGTAACCAGAAAATTGAGC<br>**reverse:** CCACTGCTGCAACACATTCATA	106

Figure S1

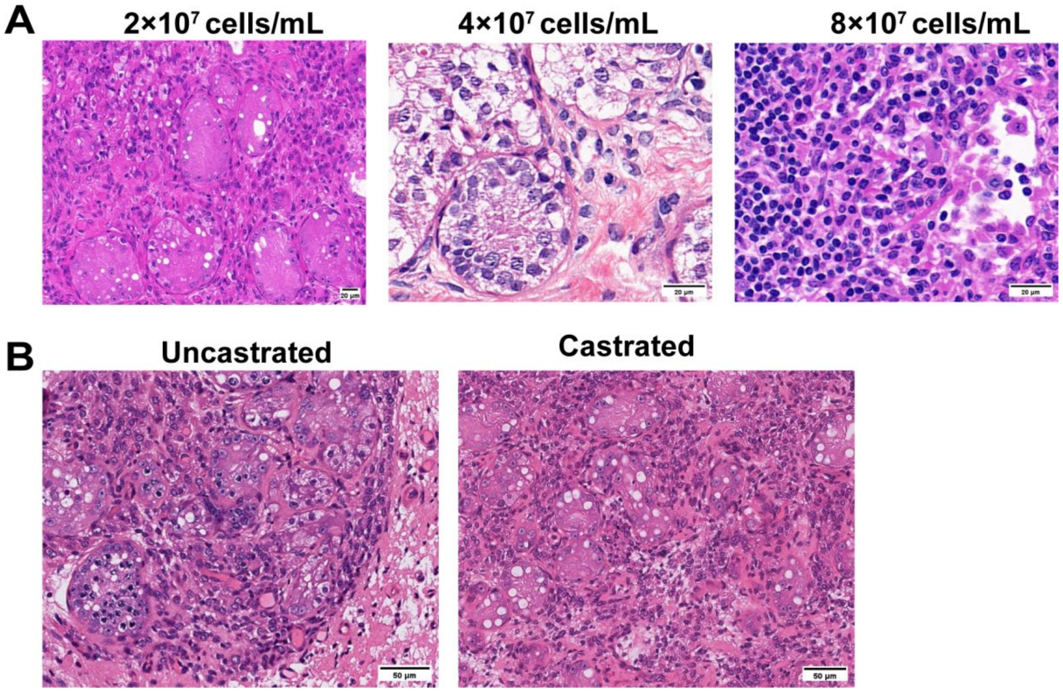

Figure S1. H&E staining of testicular organoids at 8 weeks after transplantation under different conditions. (A) Comparison under different transplantation concentration conditions. Scale bars = 20 μm. Notes: Transplantation of cell concentration: 2×10^7 cells/mL (left panel); transplantation of cell concentration: 4×10^7 cells/mL (middle panel); transplantation of cell concentration: 8×10^7 cells/mL (right panel). (B)

Comparison of castrated and uncastrated recipient nude mice. Scale bars = 50 μm.

Figure S2

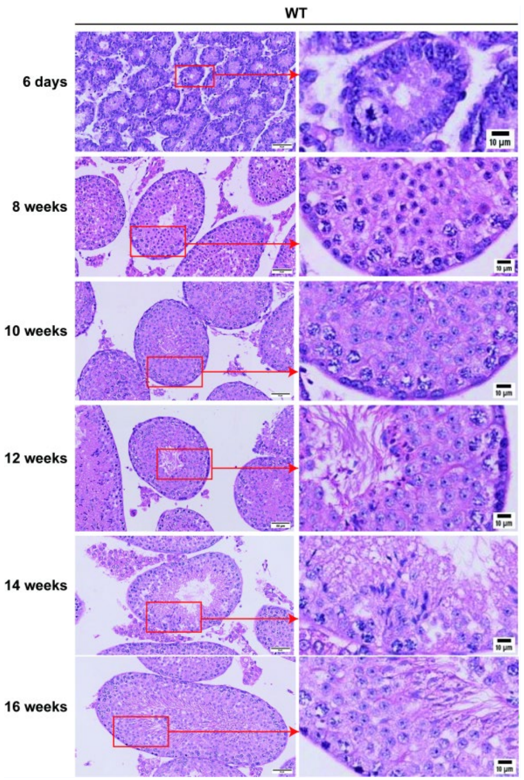

Figure S2. The morphology of normal mouse testis organs. H&E staining displayed the morphological features of 6 days, 8, 10, 12, 14, and 16

weeks of normal mouse testis organs. Scale bars in left panel=50 μm, Scale bars in right panel=10 μm.

Figure S3

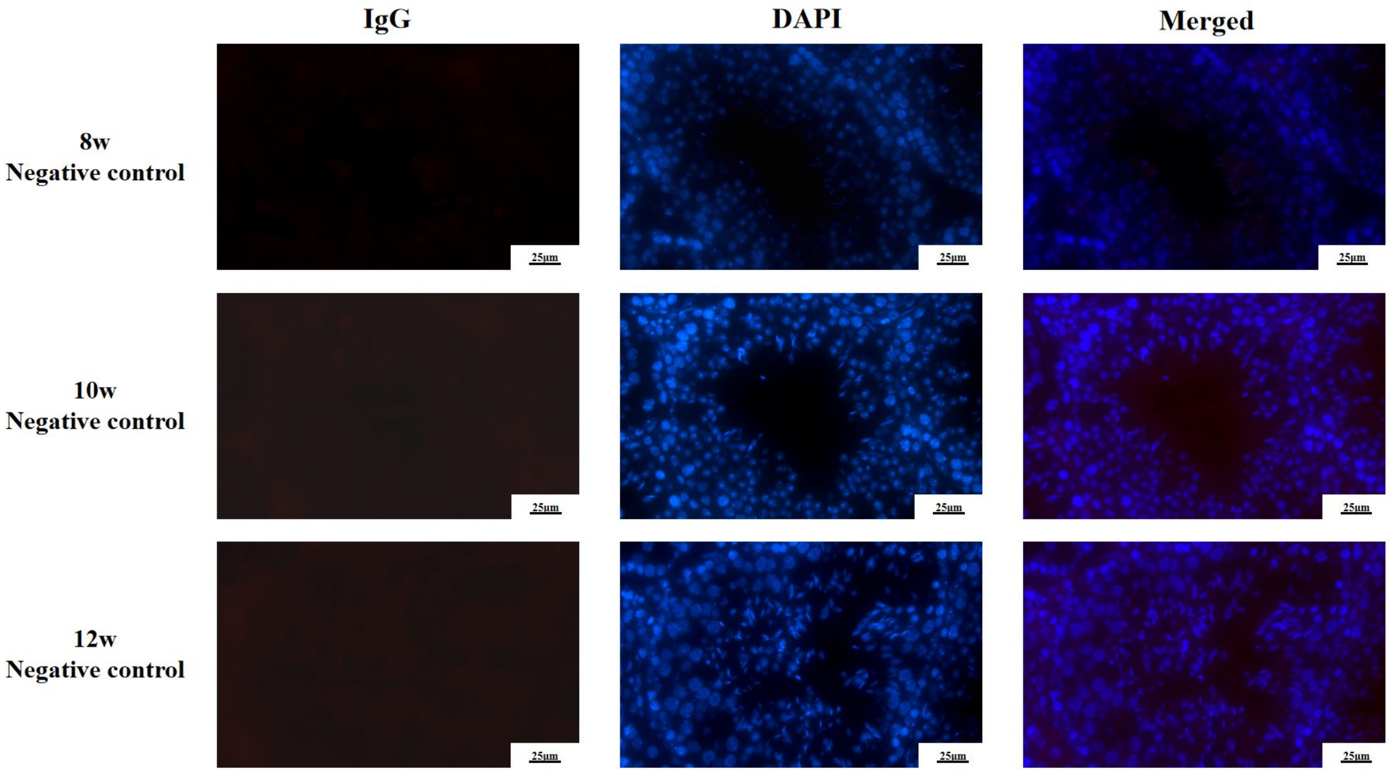

Figure S3. The expression of IgG in the mouse testicular tissues. IgG was used as a negative control for immunohistochemical staining of

normal mouse testis. Scale bars =25 μm.
